# Charantin targets HMGCR-PCSK9 axis and activates PPAR-α signaling to ameliorate hyperlipidemia: Mechanistic insights from bioinformatics and in-vivo studies

**DOI:** 10.1371/journal.pone.0331356

**Published:** 2025-09-02

**Authors:** Ghulam Abbas, Muhammad Nasir Hayat Malik, Haya Yasin, Saud O. Alshammari, Ghulam Murtaza, Waseem Hassan, Muhammad Atif, Ramla Shabbir

**Affiliations:** 1 Faculty of Pharmacy, The University of Lahore, Lahore, Punjab, Pakistan; 2 Department of Pharmaceutical Sciences, College of Pharmacy and Health Sciences, Ajman University, Ajman, United Arab Emirates; 3 Department of Pharmacognosy and Alternative Medicine, College of Pharmacy, Northern Border University, Rafha, Saudi Arabia; 4 Department of Pharmacy, COMSATS University Islamabad, Lahore, Punjab, Pakistan; Integral University, INDIA

## Abstract

Plant-derived compounds have recently gained attention owing to their better safety profile and multi-targeted actions. Charantin, a plant-based natural compound known for its diverse pharmacological properties, was investigated for its anti-hyperlipdemic activity using both in-silico and in-vivo approaches. A detailed network pharmacology analysis was used to predict charantin-related targets, cross-referenced with hyperlipidemia-associated genes from GeneCards, DisGeNET, and CTD. Shared targets were subjected to protein-protein interaction analysis and functional enrichment using STRING, Cytoscape, and ShinyGO. Molecular docking studies assessed charantin’s binding interactions with key lipid-regulating proteins (HMGCR, PCSK9, LDLR, PPAR-α, PI3K). In-vivo efficacy of charantin (100 and 200 mg/kg) was evaluated in Sprague-Dawley rats fed with high-lipid diet (HLD) for 12 days. Lipid profiles, liver enzymes and transcript levels of lipid-regulating genes were analyzed. A total of 242 overlapping genes were identified between charantin targets and hyperlipidemia-associated genes, with enrichment analyses highlighting key lipid metabolic and inflammatory pathways. Molecular docking revealed that charantin exhibited stronger binding affinities than simvastatin across multiple targets. In HLD animal model, charantin significantly reduced total cholesterol, triglycerides, LDL, and VLDL, while increasing HDL levels in a dose-dependent manner. Liver function remained preserved, accompanied by downregulation of HMGCR, PCSK9, and APOB, and upregulation of LDLR and PPAR-α at both gene and protein levels. Charantin exerts potent lipid-lowering effects through modulation of multiple pathways, including cholesterol biosynthesis, lipoprotein metabolism, and nuclear receptor activation. Its efficacy and hepatoprotective properties reiterate its potential as a safe, effective alternative or adjunct to conventional therapies for hyperlipidemia.

## Introduction

Hyperlipidemia, a metabolic disorder characterized by elevated levels of blood lipids including cholesterol, triglycerides (TG), and low-density lipoprotein (LDL), is a major modifiable risk factor for cardiovascular diseases [1,2]. The condition plays a pivotal role in the pathogenesis of atherosclerosis, coronary artery disease, and cerebrovascular accidents, contributing significantly to global morbidity and mortality [3–5]. The development of hyperlipidemia is influenced by genetic predisposition, lifestyle factors (such as excessive saturated fat intake, physical inactivity, and obesity), and metabolic comorbidities, particularly type 2 diabetes mellitus [6–10]. According to ‘WHO’ statistics, approximately 39% of the global adult population suffers from hyperlipidemia, making it a significant contributor to cardiovascular disease burden [11–13]. Since cardiovascular complications continue to be the leading cause of death worldwide, effective management of hyperlipidemia is essential to reducing cardiovascular risk and improving patient outcomes [11,14,15].

Current pharmacological interventions for hyperlipidemia include statins, fibrates, bile acid sequestrants, cholesterol absorption inhibitors, and proprotein convertase subtilisin/kexin type 9 (PCSK9) inhibitors [16–19]. Though, these treatment options demonstrate clinical efficacy in reducing lipid levels, yet present significant adverse effects including potential liver damage, muscle toxicity, gastrointestinal tract disturbances. Additionally, treatment effectiveness is often compromised by poor patient compliance resulting from prolonged therapy [20]. These challenges highlight the urgent need to develop safer, more affordable therapeutic alternatives with comparable efficacy, particularly those derived from natural compounds [21].

Charantin, a bioactive steroidal glycoside isolated from *Momordica charantia* (bitter melon), has garnered considerable attention due to its multifaceted pharmacological properties. Beyond its antidiabetic activity, charantin exhibits strong antioxidant properties by scavenging free radicals and attenuating oxidative stress, as well as anti-inflammatory effects that contribute to its cardioprotective potential [22–26]. Moreover, multiple studies have reported that *Momordica charantia* exhibits strong anti-hyperlipidemic activity, effectively reducing lipid levels and improving lipid profiles [27]. These findings suggest that charantin may play a crucial role in regulating lipid metabolism, positioning it as a promising candidate for the treatment and prevention of hyperlipidemia and associated cardiovascular conditions.

Given the limitations of current pharmacotherapies and the growing interest in plant-derived bioactive compounds, this study was designed to comprehensively investigate the lipid-lowering potential of charantin through an integrated experimental and computational approach. Utilizing a well-established animal model of hyperlipidemia, we evaluated its therapeutic efficacy in comparison to standard treatments. Furthermore, network pharmacology analysis was employed to identify potential target proteins and signaling pathways involved in charantin’s anti-hyperlipidemic effects, while molecular docking studies provided mechanistic insights into its binding interactions with key lipid-regulating enzymes and receptors. These findings not only elucidate charantin’s mode of action but also establish an evidence-based foundation for its development as either a standalone or adjunct therapy for hyperlipidemia.

## Materials and methods

### Network pharmacological analyses

#### Acquisition of charantin and hyperlipidemia targets.

The initial step involved the retrieval of charantin’s chemical structure in both SMILES and SDF formats from PubChem [28,29]. These structural files were subsequently analyzed using three target prediction platforms: PharmMapper [30,31], SuperPred [32], and WAY2DRUG [33]. The resulting protein targets were standardized and consolidated using the UniProt database to remove duplicates and ensure consistent nomenclature [34].

For ‘hyperlipidemia’ target identification, we conducted a comprehensive search across GeneCards [35], DisGeNET [36], and Comparative Toxicogenomics Database (CTD) [37] using “hyperlipidemia” as the primary search term. To enhance target specificity, GeneCards results were filtered based on median relevance scores [38]. Potential therapeutic targets were identified by intersecting the compound-related targets with disease-associated genes through Venny 2.1 software visualization. These overlapping targets, representing charantin’s potential anti-hyperlipidemic effects, were selected for subsequent pathway enrichment analysis [39].

#### Construction of protein-protein interaction network using STRING database.

To analyze the shared targets between charantin and hyperlipidemia, we performed protein-protein interaction (PPI) network analysis using the STRING database, specifically selecting “Homo sapiens” as the biological species [40,41]. The resulting network was visualized and analyzed using Cytoscape software. To identify key network components, we employed the cytoHubba plugin’s degree ranking method, which revealed the top 50 hub genes based on their connectivity within the network [42,43].

#### Functional enrichment analysis of hub genes using ShinyGO database.

The top 50 hub genes were further examined through functional enrichment analysis with ShinyGO, an online platform for Gene Ontology (GO) and Kyoto Encyclopedia of Genes and Genomes (KEGG) pathway exploration [44–47]. This analysis aimed to uncover the key biological processes and pathways associated with these genes. Using a significance threshold of p < 0.05, the 20 most statistically relevant GO terms covering biological processes (BP), cellular components (CC), and molecular functions (MF) along with the top 20 significant KEGG pathways, were identified [46–48].

### Molecular docking

#### Protein structure preparation.

The 3D crystal structures of five key proteins, low-density lipoprotein receptor (LDLR, PDB ID: 1N7D), 3-hydroxy-3-methylglutaryl-CoA reductase (HMGCR, PDB ID: 1HW8), proprotein convertase subtilisin/kexin type 9 (PCSK9, PDB ID: 6U26), peroxisome proliferator-activated receptor gamma (PPAR-α, PDB ID: 6KXY), and phosphoinositide 3-kinase (PI3K, PDB ID: 5XGI), were retrieved from the RCSB Protein Data Bank [49,50]. Using MOE software, the proteins were initially prepared by systematically removing non-essential elements such as solvent molecules, cofactors, and redundant chains. Hydrogen atoms were added, and protonation states were adjusted to reflect physiological conditions (pH 7.4). Finally, energy minimization was conducted using the AMBER10 forcefield, with a convergence threshold set at 0.1 kcal/mol/Å [51].

#### Ligand preparation and optimization.

The molecular structures of charantin (PubChem CID: 5742590) and simvastatin (PubChem CID: 54454) were acquired from PubChem in SMILES format [52]. Using MOE’s molecular modeling tools, these 2D structures were converted into optimized 3D configurations. Energy minimization was performed with the MMFF94x force field, applying an energy convergence threshold of 0.01 kcal/mol. Atomic partial charges were assigned based on the AMBER charge model. Through comprehensive conformational sampling, the lowest-energy conformers were identified and subsequently utilized for molecular docking studies [53,54].

#### Docking configuration and parameters.

Molecular docking studies were conducted using MOE’s integrated docking platform. Potential binding pockets were identified through the site finder module, which analyzes surface geometry and electrostatic properties to detect interaction sites. Docking simulations employed the ‘Triangle Matcher’ algorithm with 500 iterations for ligand placement, followed by refinement using the ‘Induced Fit’ protocol. Pose scoring was performed in two stages: initial evaluation with London dG scoring function followed by final binding energy calculations using GBVI/WSA dG. To ensure thorough conformational sampling, each ligand-protein complex underwent 50 separate docking trials. Resultant binding modes were visually analyzed across multiple visualization systems, with particular emphasis on MOE’s advanced graphical interface for detailed interaction mapping [53,55].

### In-vivo study

#### Chemicals and reagents.

Cholesterol and cholic acid were obtained from Sigma-Aldrich (St. Louis, MO, USA), while simvastatin was generously provided by Medpak Pharmaceuticals (Lahore, Pakistan). Standard diagnostic kits were procured from BioLabs (Boston, USA) and Zokeyo (Wuhan, China). Coconut oil, banaspati ghee, and all other reagents used were of analytical grade.

#### Animals.

Male Sprague-Dawley rats, 6−7 weeks old (180−200 g) were purchased from the University of Veterinary and Animal Sciences (Lahore, Pakistan). They were housed in the light-dark cycle for 12/12 hours and placed in plastic cages at 25 ± 2°C and humidity (55%) in the animal house of Faculty of Pharmacy, The University of Lahore. Prior to the experiment, all rats were acclimatized to the laboratory environment for 1 week. Experimental procedures adhered to the ARRIVE ethical guidelines and were approved by the ‘Institutional Research Ethics Committee (IREC)’ of the Faculty of Pharmacy, The University of Lahore (Approval No.: IREC-2024-18) [56,57].

#### Induction of hyperlipidemia.

Hyperlipidemia was induced by feeding rats a high-lipid diet (HLD) for 28 days. The HLD was prepared by homogenously mixing cholesterol, cholic acid, banaspati ghee and coconut oil, and egg yolk powder with standard rat chow, as described previously (Table 1) [58–60].

**Table 1:** Composition of HLD for induction of hyperlipidemia.

Components of HLD	Quantity
Cholesterol	2 g
Cholic acid	1 g
Banaspati ghee	3 g
Coconut oil	2 g
Egg yolk powder	5 g
Standard rat chow	100 g (Q.S)

On day 27, blood samples were collected to assess serum total cholesterol (TC), TG, high-density lipoprotein (HDL), LDL, and very low-density lipoprotein (VLDL) levels. Rats with TC levels >280 mg/dL were considered hyperlipidemic and selected for further experimentation [57,61,62].

Hyperlipidemic rats were subsequently treated once daily for 12 consecutive days with either simvastatin (20 mg/kg) [63] or with varying doses (100 and 200 mg/kg) of charantin. On day 40, all the animals [20] were humanely euthanized via intraperitoneal (i.p.) injection of pentobarbital sodium (200 mg/kg). Care was taken to minimize the suffering of the animals throughout the study. A well-trained laboratory technician performed all euthanasia procedures in accordance with established standard protocols. Indicators such as respiratory depression and faint heartbeat were used to determine the appropriate time for euthanasia during the study period. Importantly, no animals died spontaneously during the course of the study. Blood samples were subsequently collected for biochemical analyses, including lipid profile and liver function tests (LFTs). Liver tissues were harvested in Trizol reagent for gene expression studies. Real-time PCR was performed according to previously described method with HPRT serving as internal standard. List of primers used in this study are provided in Table 2 [57].

**Table 2:** List of primers used in this study [57].

Sr. No.	Gene name	Primers	Sequences (5’ – 3’)	BP	Tm (°C)
**1**	**LDLR**	LDLR F:	GGGTTCCATAGGGTTTCTGCT	21	60
		LDLR R:	TGGTATACTCGCTGCGGTCC	20	62
**2**	**PCSK9**	PCSK9 F:	GCACTGGAGAACCACACAGG	20	61
		PCSK9 R:	TGGCTGCATGACATTGCTTCTC	22	62
**3**	**PPAR-α**	PPAR-α F:	ACGATGCTGTCCTCCTTGATG	21	60
		PPAR-α R:	GCGTCTGACTCGGTCTTCTTG	21	61
**4**	**HPRT**	HPRT F:	CTCATGGACTGATTATGGACAGGAC	25	61
		HPRT R:	GCAGGTCAGCAAAGAACTTATAGCC	25	62
**5**	**ApoB**	APOB F:	GTAGTGGTGCGTCTTGGATC	20	58
		APOB R:	TGCAGCAAGCTGTTGAATGT	20	59
**6**	**HMGCR**	HMGCR F:	CCTCCATTGAGATCCGGAGG	20	59
		HMGCR R:	AAGTGTCACCGTTCCCACAA	20	60
**7**	**SRB1**	SRB1 F:	GGACAAGCAGTTCCAGATCCT	21	60
		SRB1 R:	GGCCAATTCAGTGTTCAGGTG	21	60

### Experimental design

Animals were randomly divided into five groups (n = 4 per group). Total number of animals used = 20

Control: Normal diet
HLD: HLD only
SIM 20 mg/kg: HLD + simvastatin (20 mg/kg)
CHTN 100 mg/kg: HLD + charantin (100 mg/kg)
CHTN 200 mg/kg: HLD + charantin (200 mg/kg)

### Statistical analysis

The data of 3–4 biological replicates were expressed as mean ± standard deviation (SD). Statistical comparisons among groups were performed using one-way analysis of variance (ANOVA), followed by Tukey’s post hoc test for multiple comparisons. Analyses were conducted using GraphPad Prism version 8.02 (GraphPad Software, Inc., San Diego, CA, USA). A p-value of less than 0.05 was considered statistically significant. The following notation was used to denote levels of significance: * p < 0.05, ** p < 0.01, *** p < 0.001.

## Results

### Network pharmacology reveals multi-target mechanisms of charantin in the regulation of hyperlipidemia

**Fig 1:**
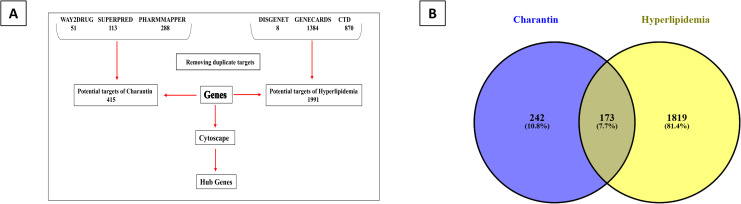
Analysis of potential therapeutic targets related to charantin. (A) Flowchart illustrating the gene identification process. (B) Venn diagram depicting 242 unique genes associated with charantin, 1,819 genes linked to hyperlipidemia, and 173 common genes that indicate overlapping pathways.

The network pharmacology analysis of charantin yielded important insights into its effects on reducing high lipid levels. Target prediction revealed 415 possible targets for charantin, while extensive database searches uncovered 1,971 genes associated with hyperlipidemia. These genes were sourced from DisGeNET (1,284 genes) and CTD (870 genes). An intersection analysis showed that 242 targets were shared between charantin and hyperlipidemia, representing 18.5% of charantin’s targets and 7.7% of the hyperlipidemia-related genes. This overlap indicates that charantin likely influences pathways related to lipid metabolism (Fig 1).

**Fig 2:**
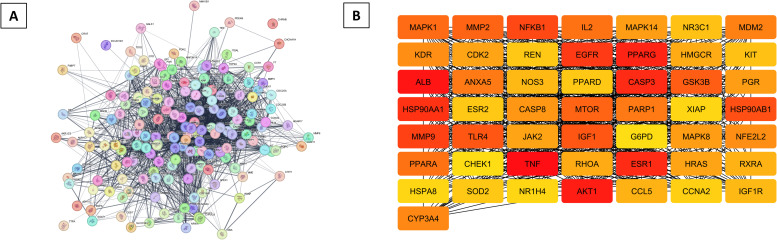
Visualization of PPI network. (A) Intricate interactions among proteins associated with charantin and hyperlipidemia (B) Top 50 hub genes identified within this network, emphasizing their importance and interconnectivity.

Using Cytoscape for PPI network analysis, 50 hub genes were identified, which are crucial in various BP. Important regulators of lipid metabolism, such as PPAR-α and PPAR-γ, along with inflammatory factors like TLR4 and COX2, and insulin signaling components including IGF1 and AKT1, were significant in this network. The hub genes listed also featured lipid regulators (HMGCR), inflammatory markers (TNF), signaling kinases (MAPK1, MAPK14, AKT1, JAK2), oxidative stress markers (NOS2, NOS3), and apoptosis-related proteins (CASP3, BAX). The PPI analysis revealed a network of 172 nodes and 2,167 edges, with an average node degree of 25.2 and a local clustering coefficient of 0.648. The expected number of interactions was 888, and the PPI enrichment p-value was < 1e-16, indicating a significant number of interactions beyond what would be expected by chance. This strong interconnectivity among the hub genes supports the idea of a multi-target mechanism of charantin (Fig 2).

These findings suggested that charantin promotes lipid breakdown mainly via PPAR signaling, modulates inflammatory pathways to decrease lipid accumulation, enhances insulin sensitivity, and reduces oxidative stress. The hub genes, particularly those involved in PPAR signaling (PPAR-α, PPAR-γ) and cholesterol metabolism (HMGCR), were the promising candidates for experimental validation and might explain why charantin was more effective at lowering lipid levels compared to traditional single-target medications. Overall, this systems-level analysis established a molecular basis for charantin’s therapeutic potential in managing hyperlipidemia, highlighting its multifaceted approach to lipid regulation and metabolic health.

### GO and KEGG analyses reveal key lipid regulatory networks targeted by charantin

GO and KEGG pathway analyses offered insights into the biological relevance of the genes identified in this study, particularly in relation to lipid metabolism and associated disorders like hyperlipidemia. The most significantly enriched term in BP category was the cellular response to lipids, underscoring the important role these genes play in lipid regulation and the cellular adaptations to changes in lipid availability. Such adaptations are vital for maintaining lipid homeostasis, which is essential for both cellular function and overall metabolic health.

Additional enriched processes involved responses to various stimuli, suggesting that these genes might participate in broader metabolic adaptations and signaling pathways that respond to changes in the cellular environment. The MF analysis revealed several key activities associated with these genes. For example, terms like nuclear receptor activity indicated that these proteins might function as transcription factors, modulating gene expression in response to lipid signals, which is particularly important for lipid metabolism as nuclear receptors are known to influence the expression of genes involved in fatty acid synthesis, oxidation, and transport. The enrichment of protein binding activities suggests that these proteins likely interact with other cellular molecules, including components of signaling pathways and lipids, further supporting their role in metabolic regulation. Predictions regarding enzyme activity indicated that these genes might catalyze important biochemical reactions related to lipid metabolism.

In the CC enrichment analysis, the identification of proteins associated with the nuclear envelope and cytoplasmic membrane highlighted their localization in critical cellular compartments for lipid processing and signaling. The nuclear envelope is essential for regulating molecular movement across the nucleus-cytoplasm interface, while the cytoplasmic membrane is crucial for lipid transport and signal transduction. This targeting suggested that the proteins were ideally positioned to directly influence lipid metabolism and respond to lipid-related signals.

**Fig 3:**
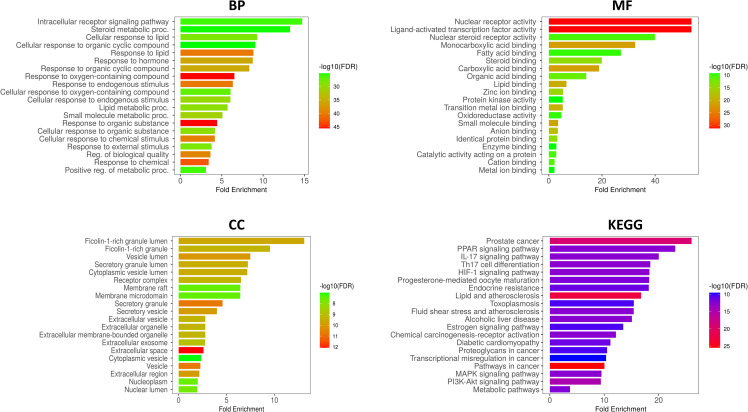
Biological function and pathway enrichment analysis of genes implicated in hyperlipidemia and charantin response. The analyses employ color codings to represent -log10(p-value), indicating the statistical significance of these findings.

KEGG pathway analysis further elucidated the specific metabolic pathways enriched among the identified genes. Notably, lipid metabolism pathways, especially fatty acid biosynthesis and lipid digestion, were highly relevant. This enrichment indicated that the genes were not only involved in lipid processing but also played a role in regulating metabolic pathways related to lipid synthesis and breakdown (Fig 3). Such findings highlight the therapeutic potential of charantin as it may target these pathways to manage lipid levels and prevent associated disorders.

### Charantin exhibits enhanced binding affinity and target modulation in lipid metabolism

The docking analysis of charantin and simvastatin against several key targets involved in lipid regulation demonstrated notable differences in binding affinities, root-mean-square deviations (RMSD), and interaction profiles, highlighting charantin’s greater potential as a therapeutic agent. For LDLR (PDB ID: 1N7D), charantin exhibited a strong binding affinity of −8.40 kcal/mol, indicating robust interactions, although it had a higher RMSD of 3.58 Å, suggesting a less stable conformation. The interactions included atom O33 of charantin bonding with residues ARG641 and PHE362 through hydrogen bonds, classified as H-acceptor and H-pi at distances of 3.19 Å and 3.75 Å, respectively. In comparison, simvastatin had a lower binding affinity of −7.22 kcal/mol and a more favorable RMSD of 1.65 Å, primarily interacting with LEU549 as an H-acceptor at a distance of 2.99 Å.

For HMGCR (PDB ID: 1HW8), charantin had a binding score of −6.75 kcal/mol and an RMSD of 2.47 Å, forming multiple hydrogen bonds with residues GLU700, THR636, and LYS606, involving both H-donor and H-acceptor interactions at distances between 2.83 Å and 3.08 Å. In contrast, simvastatin displayed a binding affinity of −6.08 kcal/mol with a more stable RMSD of 0.89 Å, engaging with ILE638 as an H-acceptor at a distance of 3.08 Å, indicating a better docking conformation despite its lower binding affinity. For PCSK9 (PDB ID: 6U26), charantin achieved a binding score of −7.84 kcal/mol with an RMSD of 2.49 Å, involving atoms O33 and O36 and residues ASN439 and THR459 through H-acceptor interactions at distances of 3.02 Å and 3.33 Å. Simvastatin had a binding score of −7.73 kcal/mol and a higher RMSD of 1.96 Å, interacting with TRP461 as an H-acceptor at a distance of 2.90 Å. Regarding the PPAR-α (PDB ID: 6KXY), charantin showed a binding affinity of −7.00 kcal/mol and an RMSD of 1.79 Å, interacting with residues TYR334, GLY335, and ASN219 through multiple H-acceptor interactions at distances ranging from 2.76 Å to 3.12 Å. Simvastatin had a binding affinity of −7.23 kcal/mol with a stable RMSD of 1.5593 Å, engaging with MET355 as an H-donor at a distance of 3.47 Å.

Moreover, for PI3K (PDB ID: 5XGI), charantin achieved a binding score of −7.60 kcal/mol and an RMSD of 1.66 Å, interacting with ASP806 as an H-donor at a distance of 2.86 Å. Simvastatin exhibited a binding affinity of −7.37 kcal/mol and a higher RMSD of 3.1781 Å, with interactions involving GLY1009 and GLN1014 through H-donor and H-acceptor mechanisms.

**Fig 4:**
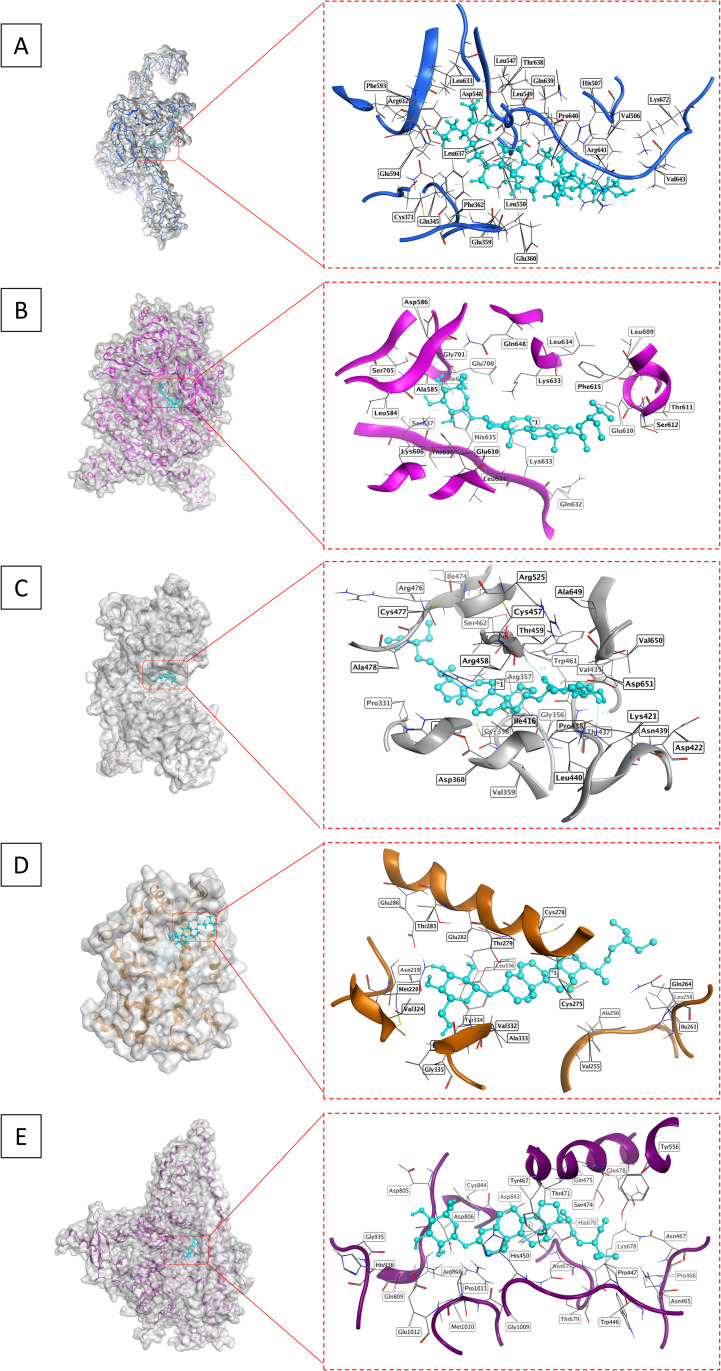
Structural analysis of charantin’s binding interactions with key lipid regulating targets. (A) LDLR shown in blue; (B) HMGCR depicted in magenta; (C) PCSK9 in grey; (D) PPAR-α in orange; and (E) PI3K represented in purple. Each target is presented with a close-up view of the binding site, emphasizing the interactions with charantin.

**Fig 5:**
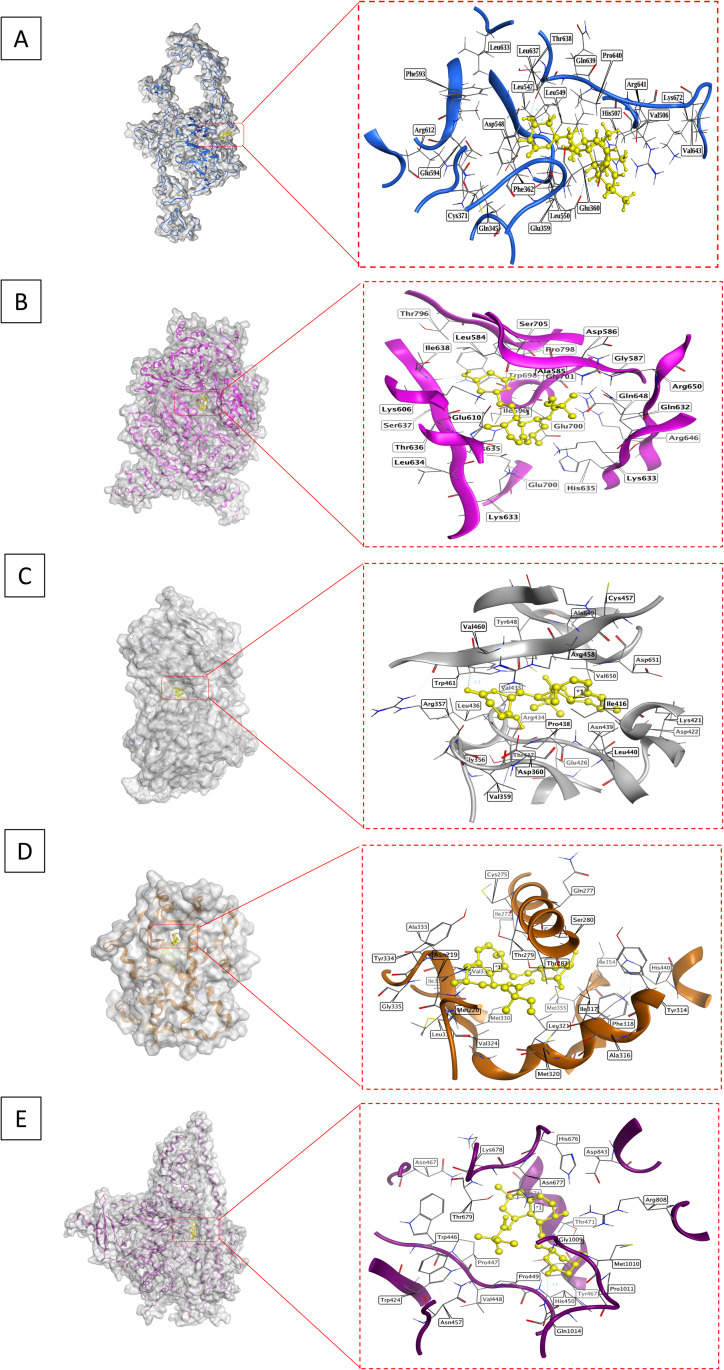
Interaction profiles of simvastatin with key proteins involved in lipid regulation. (A) LDLR shown in blue; (B) HMGCR depicted in magenta; (C) PCSK9 in grey; (D) PPAR-α in orange; and (E) PI3K represented in purple. Each target is presented with a close-up view of the binding site, emphasizing the interactions with simvastatin.

Overall, these results indicate that charantin consistently shows stronger binding affinities across multiple targets compared to simvastatin. The binding energies, represented as S scores, suggest that charantin has a higher affinity for these targets, which could potentially lead to enhanced biological efficacy. Charantin’s scores ranged from −8.4020 to −6.7559 kcal/mol, whereas simvastatin’s ranged from −6.0851 to −7.7303 kcal/mol. Although charantin presented higher RMSD values in some instances (up to 3.5829 Å for LDLR), it maintained favorable binding energies. In some instances, charantin’s RMSD was comparable to or lower than that of simvastatin, indicating greater stability. The interaction profiles further clarify the nature and specificity of the binding interactions, with charantin forming multiple hydrogen bonds with various residues across the targets. These extensive interactions not only enhance binding affinity but also suggest effective target’s modulation. While simvastatin demonstrated effective interactions, it did not achieve the same breath as charantin. The superior binding affinities, favorable RMSD values, and extensive interaction profiles of charantin underscore its potential as a more effective therapeutic agent than simvastatin for managing lipids and associated disorders (Table 3, Figs 4 and 5).

**Table 3:** Molecular docking analysis of charantin and simvastatin with different proteins.

Compounds	S Score (kcal/mol)	RMSD (Å)	Atom of compounds	Atom of receptors	Residue of receptor	Type of interactions	Distance (Å)	*E* (kcal/mol)
**LDLR (PDB ID: 1N7D)**								
**Charantin**	−8.4020	3.5829	O33 C7	NH2 6-ring	ARG 641 (A) PHE 362 (A)	H-acceptor H-pi	3.19 3.75	−1.7 −0.7
**Simvastatin**	−7.2208	1.6539	O29	N	LEU 549 (A)	H-acceptor	2.99	*−2.0*
**HMGCR (PDB ID: 1HW8)**								
**Charantin**	−6.7559	2.4723	O34 O36 O35	O O NZ	GLU 700 (C) THR 636 (B) LYS 606 (B)	H-donor H-donor H-acceptor	2.88 2.83 3.08	−1.1 −2.5 −4.2
**Simvastatin**	−6.0851	0.8911	O29	N	ILE 638 (B)	H-acceptor	3.08	−2.2
**PCSK9 (PDB ID: 6U26)**								
**Charantin**	−7.8434	2.4912	O33 O36	N N	ASN 439 (B) THR 459 (B)	H-acceptor H-acceptor	3.02 3.33	−2.2 −0.8
**Simvastatin**	−7.7303	1.9678	O27	N	TRP 461 (B)	H-acceptor	2.90	−3.1
**PPAR-α (PDB ID: 6KXY)**								
**Charantin**	−7.0035	1.7972	O31 O33 O35	N N ND2	TYR 334 (A) GLY 335 (A) ASN 219 (A)	H-acceptor H-acceptor H-acceptor	2.76 3.12 2.90	−3.3 −0.9 −1.6
**Simvastatin**	−7.2374	1.5593	C23	SD	MET 355 (A)	H-donor	3.47	−0.5
**PI3K (PDB ID: 5XGI)**								
**Charantin**	−7.6082	1.6671	O36	OD2	ASP 806 (A)	H-donor	2.86	−3.7
**Simvastatin**	−7.3705	3.1781	O29 O27	O NE2	GLY 1009 (A) GLN 1014 (A)	H-donor H-acceptor	2.81 3.16	−2.8 −1.6

### Charantin attenuates hyperlipidemia through modulation of TC, TG, HDL, and VLDL: A comparative study with simvastatin

In in-vivo study, the HLD groups exhibited elevated TC levels and treatment with simvastatin (20 mg/kg) significantly reduced TC, demonstrating its efficacy as a reference lipid-lowering agent. Both doses of charantin (100 and 200 mg/kg) also produced a marked reduction in TC, with the higher dose (200 mg/kg) showing a more pronounced effect, suggesting a dose-dependent response.

A similar trend was observed for TG, where the HLD group displayed the highest levels, indicative of hypertriglyceridemia. Simvastatin effectively lowered TG, while charantin at both doses also reduced TG levels, with the 200 mg/kg dose achieving a more substantial decrease.

**Fig 6:**
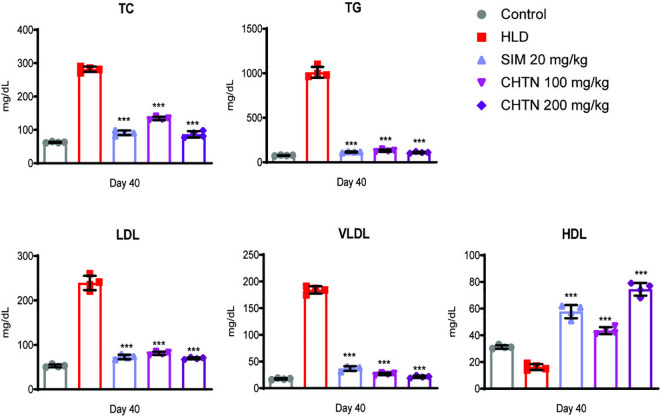
Effects of charantin and simvastatin on serum lipid profiles in hyperlipidemic rat model. Serum levels of TC, TG, LDL, VLDL and HDL were measured at day 40. Charantin treatment dose-dependently improved lipid profiles, with the 200 mg/kg dose exhibiting efficacy superior or comparable to SIM. Significant differences are indicated by asterisks, with ***p < 0.001, **p < 0.01, and *p < 0.05, highlighting the effects of charantin and simvastatin on lipid profile compared to the HLD group.

HDL levels, often referred to as “good cholesterol,” were notably lowered in the HLD group compared to the control. Treatment with simvastain and both charantin doses resulted in an increase in HDL, with the higher charantin dose (200 mg/kg) showing a more significant improvement, aligning with its potential cardioprotective effects. VLDL levels, which correlate with triglyceride metabolism, were elevated in the HLD group. Simvastatin and charantin treatments reduced VLDL concentrations, with the 200 mg/kg charantin dose demonstrating the most effective reduction, further supporting its role in improving lipid metabolism (Fig 6).

The results collectively indicate that charantin, particularly at the 200 mg/kg dose, exhibited comparable or superior lipid-modulating effects relative to simvastatin, highlighting its potential as a therapeutic agent for managing dyslipidemia. The dose-dependent responses observed for charantin underscore its efficacy in ameliorating lipid abnormalities.

### Charantin alleviates hyperlipidemia-induced hepatic injury: Evidence from ALT and AST biomarker modulation

**Fig 7:**
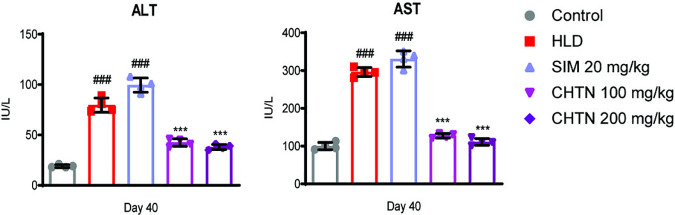
Effects of charantin and simvastatin on serum liver enzymes in hyperlipidemic rat model. HLD and SIM displayed a significant rise in AST and ALT levels while, charantin dose dependently attenuated the levels of liver enzymes. Significant differences are indicated by asterisks, with ***p < 0.001, **p < 0.01, and *p < 0.05, highlighting the effects of charantin and SIM on lipid profile compared to the HLD group. ###p < 0.001 indicates comparison of HLD and SIM groups with untreated control group.

In order to assess the hepatoprtective effects of CHTN, serum alanine aminotransferase (ALT) and aspartate aminotransferase (AST) levels across experimental groups were measured. The HLD group showed substantially elevated ALT and AST levels compared to the Control group, reflecting significant hyperlipidemia-induced hepatic injury. Treatment with SIM (20 mg/kg) resulted in a notable induction in both ALT and AST levels, consistent with its established hepatotoxic properties. CHTN administration demonstrated a clear dose-dependent response, with the 100 mg/kg dose producing a moderate decrease in liver enzyme levels and the 200 mg/kg dose exhibiting a more pronounced reduction. These findings suggest that CHTN, particularly at the higher dose, effectively mitigates liver damage associated with hyperlipidemia, likely through mechanisms such as anti-inflammatory activity, antioxidant effects, or modulation of lipid metabolism. The results highlight the therapeutic potential of CHTN as a hepatoprotective agent, with its efficacy at 200 mg/kg supporting further investigation into its underlying mechanisms (Fig 7).

### Charantin ameliorates dyslipidemia by targeting PCSK9, HMGCR, PPAR-α, and LDLR expression in HLD-induced rats

In order to validate the in-silico findings, the relative gene expression profiles of PCSK9, HMGCR, PPAR-α, SRB1, LDLR, and APOB were measured across five experimental groups: Control, HLD, simvastatin-treated (SIM, 20 mg/kg), and charantin-treated groups (CHTN 100 mg/kg and CHTN 200 mg/kg).

A significant upregulation of PCSK9 expression was observed in the HLD group compared to the control, indicating enhanced degradation of LDLR and impaired cholesterol clearance. Treatment with both doses of charantin, resulted in a marked downregulation of PCSK9 expression (p < 0.001), suggesting a potential mechanism for improved lipid homeostasis through the preservation of LDLR activity.

Expression of HMGCR, the rate-limiting enzyme in cholesterol biosynthesis, was also significantly elevated in the HLD group. Simvastatin treatment effectively suppressed HMGCR expression (p < 0.001), consistent with its known pharmacological action. Similarly, both doses of charantin significantly reduced HMGCR expression (p < 0.01 and p < 0.001 for 100 and 200 mg/kg, respectively), with a dose-dependent trend, indicating its potential inhibitory effect on endogenous cholesterol synthesis.

PPAR-α, a nuclear receptor involved in fatty acid oxidation, was downregulated in the HLD group. Charantin treatment significantly enhanced PPAR-α expression in a dose-dependent manner (p < 0.05 for 100 mg/kg; p < 0.001 for 200 mg/kg), whereas simvastatin showed no significant effect. The pronounced upregulation observed with CHTN 200 mg/kg indicates activation of lipid catabolic pathways.

Expression of SRB1, a receptor involved in reverse cholesterol transport, remained unchanged in the HLD and simvastatin groups. However, treatment with charantin at 200 mg/kg significantly increased SRB1 expression (p < 0.001), suggesting enhanced cholesterol efflux and HDL-mediated transport.

LDLR expression was modestly increased in the HLD group and further upregulated following treatment, with the highest expression observed in the CHTN 200 mg/kg group (p < 0.001). These findings imply improved LDL clearance through upregulation of hepatic LDLR, particularly with higher charantin dosage.

**Fig 8:**
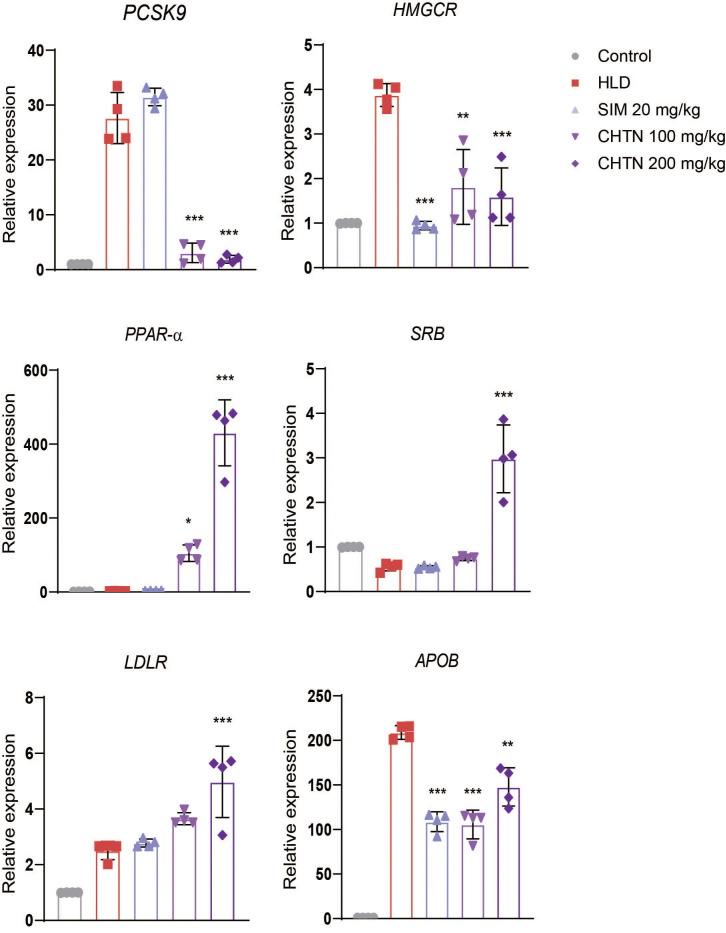
Charantin differentially regulate lipid-metabolism related genes. The bar graphs depict the relative expression levels of key lipid metabolism biomarkers (PCSK9, HMGCR, PPAR-α, SRB1, LDLR, and APOB) in control, high lipid diet (HLD), simvastatin (SIM), and charantin (CHTN) treated groups. Significant differences are indicated by asterisks, with ***p < 0.001, **p < 0.01, and *p < 0.05, highlighting the effects of charantin and SIM on gene expression compared to the HLD group.

Finally, APOB, a key structural protein of LDL particles, was markedly upregulated in the HLD group. Both simvastatin and charantin significantly attenuated APOB expression, with greater reductions observed in the CHTN 200 mg/kg group (p < 0.01), suggesting suppression of atherogenic lipoprotein synthesis (Fig 8).

**Fig 9:**
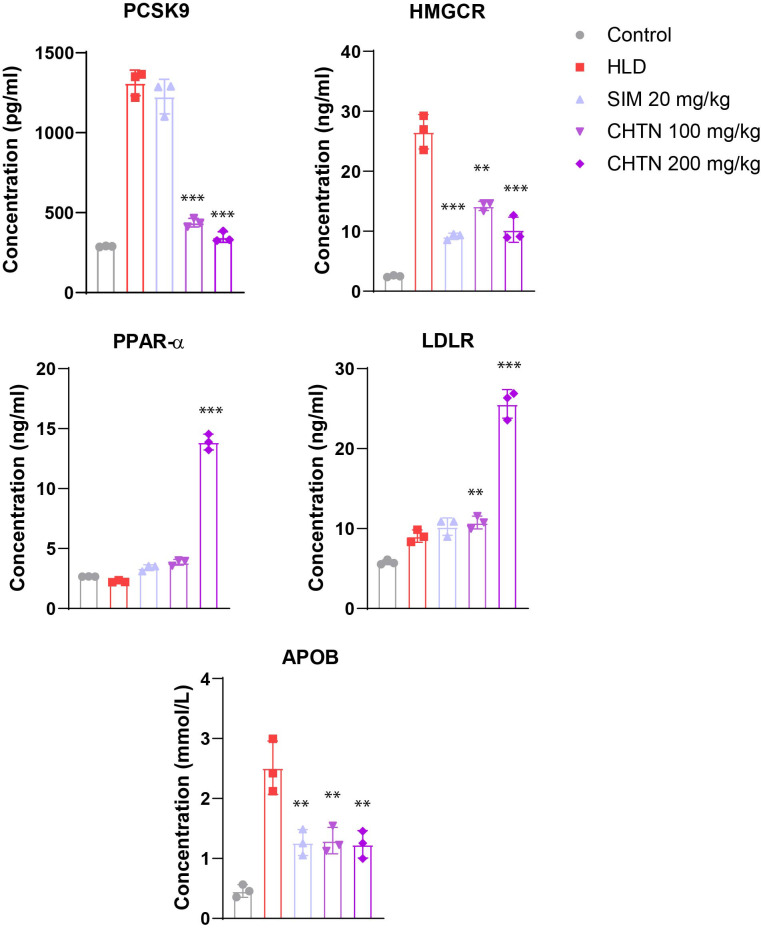
ELISA-based validation of charantin’s modulatory effects on lipid-regulatory proteins in hyperlipidemic rats. Charantin displayed a dose-dependent reduction in HMGCR, PCSK9, APOB levels along with an induction in PPAR-α and LDLR levels. Significant differences are indicated by asterisks, with ***p < 0.001 and **p < 0.01 highlighting the effects of charantin and simvastatin on gene expression compared to the HLD group.

ELISA analysis also reiterated the beneficial effects of charantin in the regulation of lipid metabolism. Like gene expression studies, PCSK9 protein levels were raised in HLD group when compared to the control group. CHTN at doses of 100 mg/kg and 200 mg/kg resulted in significant reductions in PCSK9 levels, with p-values less than 0.001. Similar trends were observed for HMGCR, where the control group had the lowest levels, while the HLD group displayed increased concentrations of approximately 800 ng/ml. Treatments with SIM and CHTN led to a significant reduction (p < 0.001). PPAR-α levels were markedly elevated in the HLD group, reaching around 600 ng/ml, but were significantly decreased by both SIM (p < 0.001) and CHTN at both doses (p < 0.01 for 100 mg/kg and p < 0.001 for 200 mg/kg). Likewise, LDLR levels were significantly higher in the HLD group compared to the control, with CHTN treatments significantly increasing LDLR concentrations (p < 0.01 for CHTN 100 mg/kg and p < 0.001 for both SIM and CHTN 200 mg/kg). Finally, APOB levels were elevated in the HLD group at approximately 0.5 mmol/L, but treatment with SIM and CHTN resulted in significant reductions (p < 0.01 for SIM and CHTN 200 mg/kg, and p < 0.05 for CHTN 100 mg/kg) (Fig 9).

Overall, the results demonstrate that charantin exerts dose-dependent modulatory effects on lipid metabolism-related genes, with efficacy comparable to or greater than simvastatin in several pathways, supporting its potential as a therapeutic agent for hyperlipidemia.

## Discussion

This study provided a meticulous assessment of charantin’s anti-hyperlipidemic effect via in-silico and in-vivo analyses. The combination of network pharmacology, molecular docking, and in-vivo experimental techniques provided a thorough understanding of charantin’s lipid-modulating characteristics, making it a viable lead for hyperlipidemia management.

Network pharmacology analysis revealed that charantin interacted with 173 targets related to hyperlipidemia, highlighting its broad pharmacological spectrum. This multi-targeted response is beneficial in case of complex metabolic diseases which include numerous pathways, such as hyperlipidemia [40,64]. The PPI network demonstrated a higher degree of interconnectivity among charantin’s targets. These findings are consistent with new pharmacological perspectives that highlight the benefits of multi-target therapies over single-target medications in the treatment of complex illnesses [20,65]. The enrichment analyses further strengthened these observations, demonstrating significant involvement of lipid-responsive pathways, nuclear receptor activity and cellular response to lipids. Together, these results suggest that the therapeutic potential of charantin is derived from its capability to regulate many lipid homeostasis processes, such as synthesis, oxidation and transport [66].

Molecular docking studies provided new insights into charantin’s interactions with key lipid-regulating proteins, including HMGCR, PCSK9, LDLR, PPAR-α,. Although simvastatin showed more favorable RMSD values in some cases, yet charantin exhibited superior binding affinities, suggesting more stable interactions at target sites [22]. These interactions likely contribute to its pharmacological effects by modulating receptor activity and enzyme function.

In-silico data were further substantiated by in-vivo experimentation, which demonstrated charantin’s dose-dependent lipid-lowering effects in hyperlipidemic rats. In comparison to simvastatin, charantin displayed comparable or superior efficacy in reducing TC, TG, LDL, VLDL while increasing HDL levels. These findings align with anti-hyperlipidemic studies of *Momordica charantia*, however, they provide novel mechanistic insights through comprehensive gene expression analysis [23]. The improvement in lipid profiles was also accompanied with hepatoprotective effects, as evidenced by reduced serum ALT and AST levels, suggesting that charantin may overcome the hepatotoxicity associated with some conventional lipid-lowering drugs [63,67]. This safety profile is particularly relevant for long-term hyperlipidemia management, where drug-induced liver injury remains a major concern [68].

Gene expression analysis provided the probable mechanism behind charantin’s observed effects. In our study, hyperlipidemic rats exhibited significant upregulation of HMGCR, which was markedly reversed by charantin in a dose-dependent fashion. The suppression of HMGCR expression by charantin indicates that it may share similar cholesterol inhibitory effects like statins. HMGCR catalyzes the conversion of HMG-CoA to mevalonate, an essential step in cholesterol biosynthesis [65,69]. Inhibiting this enzyme is the primary mechanism for statins, which significantly lower cholesterol levels and risk of cardiovascular events [70]. Charantin’s lipid-modulating profile was further enhanced by its regulatory action on APOB, the main apolipoprotein of chylomicrons, VLDL and LDL particles [70]. Studies indicate that the secretion of atherogenic lipoproteins is highly dependent on APOB levels and elevated level of this protein is correlated with increase incidences of cardiovascular disorders [71,72]. Higher levels of APOB signify more LDL particles, which more accurately predict atherosclerosis risk than LDL alone [63,73]. In our study, charantin significantly downregulated APOB expression in hyperlipidemic rats, suggesting reduced hepatic secretion of VLDL and improved clearance of LDL particles.

Another key target in the regulation of lipid homeostasis is PCSK9, which mediates the degradation of hepatic LDLR and thereby attenuates LDL clearance [38,74]. LDLR facilitates hepatic clearance of circulating LDL and is a key determinant of plasma LDL levels [64,75]. Elevated PCSK9 levels are associated with hypercholesterolemia, particularly in familial cases and PCSK9 inhibitors have recently garnered attention due to their effectiveness in statin-intolerant populations [11,76,77]. Our study revealed that charantin significantly downregulated PCSK9 gene expression. Moreover, a significant upregulation in LDLR transcript levels was witnessed, suggesting enhanced receptor-mediated endocytosis of LDL. This aligns with prior reports that natural compounds, such as berberine and resveratrol, improve lipid profiles via LDLR upregulation [11,66].

One of the most compelling findings in this study was charantin’s influence on PPAR-α, a nuclear receptor that governs the transcription of genes involved in β-oxidation [66,78]. PPAR-α activation reduces TG-rich lipoproteins and improves HDL levels by enhancing hepatic lipid metabolism [6,79]. In contrast to simvastatin, which did not significantly alter PPAR-α expression, charantin induced a prominent dose-dependent upregulation. This suggests that charantin may mimic the lipid-lowering effects of classical PPAR-α agonists, i.e., fibrates [32]. Importantly, PPAR-α activation also exerts anti-inflammatory and anti-oxidant effects, contributing to its cardioprotective effect [80,81]. Given that oxidative stress and inflammation are central to the development of atherosclerosis [82], charantin’s modulating effect on PPAR-α offers a new dimension to its therapeutic potential [64].

Collectively, these findings highlight charantin’s multifactorial mechanism of action in lipid regulation. By simultaneously targeting multiple pathways including inhibition of cholesterol synthesis via HMGCR, reduction of lipoprotein secretion via APOB, preservation of LDLR through PCSK9 suppression, enhancement of LDLR expression and activation of lipid oxidation through PPAR-α, charantin offers a holistic approach to managing dyslipidemia.

## Conclusion

In conclusion, the current study provides compelling evidence that charantin exerts potent anti-hyperlipidemic effects through simultaneous modulation of critical targets such as HMGCR, APOB, PCSK9, PPAR-α and LDLR. Its favorable docking interactions, gene expression modulation, and hepatoprotective effects suggest that it may serve as a promising alternative or adjunct to current lipid-lowering therapies, especially in patients intolerant to statins or with complex metabolic disorders. Future studies should explore the long-term safety and efficacy of charantin in larger animal models and human trials and investigate its effects on lipid metabolism under different dietary and genetic backgrounds.
